# Phthalate and phthalate replacement concentrations in relationship to adiposity in a multi-racial cohort of children

**DOI:** 10.1038/s41366-024-01548-w

**Published:** 2024-06-01

**Authors:** Kelly J. Hunt, Pamela L. Ferguson, Michael S. Bloom, Brian Neelon, John Pearce, Sarah Commodore, Roger B. Newman, James R. Roberts, Lisa Bain, William Baldwin, William A. Grobman, Anthony C. Sciscione, Alan T. Tita, Michael P. Nageotte, Kristy Palomares, Daniel W. Skupski, Cuilin Zhang, Ronald Wapner, John E. Vena

**Affiliations:** 1 Department of Public Health Sciences, Medical University of South Carolina, Charleston, SC USA; 2 Department of Global and Community Health, George Mason University, Fairfax, VA USA; 3 Department of Environmental and Occupational Health, Indiana University, Bloomington, IN USA; 4 Department of Obstetrics and Gynecology, Medical University of South Carolina, Charleston, SC USA; 5 Department of Pediatrics, Medical University of South Carolina, Charleston, SC USA; 6 Department of Biological Sciences, Clemson, SC USA; 7 Department of Obstetrics and Gynecology, The Ohio State University Wexner Medical Center, Columbus, OH USA; 8 Department of Obstetrics and Gynecology, Christiana Care Health System, Newark, DE USA; 9 Department of Obstetrics and Gynecology and Center for Women’s Reproductive Health, University of Alabama at Birmingham, Birmingham, AL USA; 10 Department of Obstetrics and Gynecology, Miller Children’s and Women’s Hospital, Long Beach, CA USA; 11 Department of Obstetrics and Gynecology, Saint Peter’s University Hospital, New Brunswick, NJ USA; 12 Department of Obstetrics and Gynecology, New York Presbyterian Queens Hospital, Queens, NY USA; 13 Eunice Kennedy Shriver National Institute of Child Health and Human Development, National Institutes of Health, Bethesda, MD USA; 14 Global Center for Asian Women’s Health and Department of Obstetrics & Gynaecology, Yong Loo Lin School of Medicine, National University of Singapore, Singapore, Singapore; 15 Department of Obstetrics and Gynecology, Columbia University Medical Center, New York, NY USA

**Keywords:** Risk factors, Obesity

## Abstract

**Background/Objective:**

Phthalates and phthalate replacements are used in multiple everyday products, making many of them bioavailable to children. Experimental studies suggest that phthalates and their replacements may be obesogenic, however, epidemiologic studies remain inconsistent. Therefore, our objective was to examine the association between phthalates, phthalate replacements and childhood adiposity/obesity markers in children.

**Subjects/Methods:**

A cross-sectional study was conducted in 630 racial/ethnically diverse children ages 4–8 years. Urinary oxidative metabolites of DINCH and DEHTP, three low molecular weight (LMW) phthalates, and eleven high molecular weight (HMW) phthalates were measured. Weight, height, waist circumference and % body fat were measured. Composite molar sum groups (nmol/ml) were natural log-transformed. Linear regression models adjusted for urine specific gravity, sex, age, race-ethnicity, birthweight, breastfeeding, reported activity level, mother’s education and pre-pregnancy BMI.

**Results:**

All children had LMW and HMW phthalate metabolites and 88% had DINCH levels above the limit of detection. One unit higher in the log of DINCH was associated with 0.106 units lower BMI *z*-score [*β* = −0.106 (95% CI: −0.181, −0.031)], 0.119 units lower waist circumference *z*-score [*β* = −0.119 (95% CI: −0.189, −0.050)], and 0.012 units lower percent body fat [*β* = −0.012 (95% CI: −0.019, −0.005)]. LMW and HMW group values were not associated with adiposity/obesity.

**Conclusions:**

We report an inverse association between child urinary DINCH levels, a non-phthalate plasticizer that has replaced DEHP in several applications, and BMI *z*-score, waist circumference *z*-score and % body fat in children. Few prior studies of phthalates and their replacements in children have been conducted in diverse populations. Moreover, DINCH has not received a great deal of attention or regulation, but it is a common exposure. In summary, understanding the ubiquitous nature of these chemical exposures and ultimately their sources will contribute to our understanding of their relationship with obesity.

## Introduction

The prevalence of obesity in youth worldwide has more than tripled in the past 40 years despite targeted efforts to curb the epidemic [1]. Childhood obesity is associated with poor cardiovascular risk profiles that track into adulthood and compound an individual’s total cardiovascular burden early in life. Obesity disproportionally affects non-Hispanic Black (20%) and Hispanic (24%) youth when compared to non-Hispanic White (15%) and Asian (10%) youth [1]. Studies have also indicated that body composition and fat storage differs across race and ethnic groups with Asian and Asian Americans having increased central adiposity at lower body mass index (BMI) levels [2].

Phthalates and phthalate replacements are used in multiple everyday products, making many of them bioavailable to children. Low molecular weight (LMW) phthalates are used in personal-care products (perfumes, lotions, cosmetics), solvents, plasticizers, lacquers, varnishes, coatings, and timed releasers in some pharmaceuticals [3]. High molecular weight (HMW) phthalates are plasticizers found in flexible vinyl used in consumer products, flooring, wall coverings, food packaging, and medical devices [3]. Diethylhexyl phthalate (DEHP) is the most common member of the phthalate class. Di-2-ethylhexyl terephthalate (DEHTP) is a structural isomer of DEHP and often used as a replacement for DEHP which is increasingly regulated. Di(isononyl) cyclohexane-1,2-dicarboxylate (DINCH) is a non-phthalate plasticizer that has replaced DEHP and other HMW phthalates in several applications [4]. These chemicals are ubiquitous in the environment and shed into household dust. Children are especially at risk of exposure to HMW phthalates because of their propensity to touch many things and place their hands in their mouths [4, 5].

Evidence concerning the effects of phthalates and phthalate replacements on child adiposity is inconsistent. Phthalates are known for their endocrine disruption properties with prior studies reporting inconsistent sex-specific differences in relationship to adiposity/obesity outcomes [6–10]. Moreover, while studies of phthalates have included diverse cohorts, race-ethnic specific differences between phthalate/phthalate replacements and obesity have not been previously reported. Viewing race and ethnicity as social constructs, the motivation for examining race-ethnic specific differences stems from potential social and environmental differences.

Experimental evidence suggests that phthalates and phthalate replacements may be obesogenic [11], however, epidemiologic studies of the effects of phthalates and phthalate replacements on child adiposity remain inconsistent overall and with respect to sex-specific differences, and few studies have compared racially and ethnically diverse participants [12]. Therefore, this study examines the association between urinary levels of phthalate and phthalate replacements and childhood adiposity/obesity markers in a diverse cohort of children ages 4 to 8 years. In total 16 metabolites were examined, including 14 phthalate metabolites and two metabolites of DINCH a phthalate replacement. Additionally, interactions between phthalates and phthalate replacements and sex as well as race-ethnic group were examined for each of the outcomes of interest.

## Materials/subjects and methods

### Study population

The current study is based on data from participants enrolled in the *Eunice Kennedy Shriver* National Institute of Child Health and Human Development (NICHD) Fetal Growth Studies—Singletons cohort. That study enrolled 2802 women ages 18–40 at 12 U.S. clinical centers (2009–2013) between 8 and 13 weeks of gestation [13]. Because the primary aim of the NICHD Fetal Growth Studies was to develop fetal growth standards, enrollment was restricted to women without preexisting chronic diseases, medical conditions, or obesity (*n* = 2334) [13]. Secondary aims were to examine the etiology of gestational diabetes and associations of obesity on fetal growth and a supplemental cohort of women with obesity was recruited (*n* = 468) [14].

The Environmental Influences on Child Health Outcomes (ECHO-FGS) study consists of mother-child pairs recruited from May 2017 through April 2019 who had originally participated in the NICHD Fetal Growth Studies cohort at ten of the original 12 sites. These sites had enrolled 2373 women who remained in Fetal Growth Studies through delivery. ECHO-FGS study participants with phthalates measured (*n* = 630) in children were similar to other Fetal Growth Studies participants (*n* = 1743) with respect to child sex, birth year, maternal age and education level (all *p* values > 0.15). In contrast, mothers of children with phthalates measured were more likely to identify as NHW or NHB and less likely to identify as Hispanic or Asian/Pacific Islander compared to the other Fetal Growth Studies participants (*p* value < 0.0001). Written informed consent was obtained from the parent or legal guardian of each child. Depending on child age and state regulation child assent was obtained. The study was approved by the Medical University of South Carolina Institutional Review Board (IRB) and a Central IRB at Columbia University Medical Center and by all the participating sites and all methods were performed in accordance with the relevant guidelines and regulations.

### Child’s urine analysis

A single spot urine sample was collected from each child and the analysis of urine collected from children was performed masked to all child characteristics at NSF International (Ann Arbor, MI, USA) by LC-MS-MS using a Thermo Scientific Transcend TXII Turbulent Flow system interfaced with a Thermo Scientific Quantiva triple quadrupole mass spectrometer using multiple reaction monitoring in negative mode for sixteen different phthalates. The method was developed to replicate The Centers for Disease Control and Prevention (CDC) Phthalate Metabolites in Urine Method No: 6306.03, Revised: July 3, 2010 [15]. The NSF method was evaluated against acceptance criteria established within the CDC method. Sixteen metabolites were measured including three metabolites of LMW phthalates (monoethyl phthalate (MEP), mono-isobutyl phthalate (MIBP), mono-n-butyl phthalate (MNBP)), eleven metabolites of HMW phthalates (monobenzyl phthalate (MBZP), mono-ethylhexyl-phthalate (MEHP), monoisononyl phthalate (MINP), mono-(2-ethyl-5-oxohexyl) phthalate (MEOHP), mono-(2-ethyl-5-carboxypentyl) phthalate (MECPP), mono-(2-ethyl-5-hydroxyhexyl) phthalate (MEHHP), mono-carboxy isooctyl phthalate (MCIOP), mono-carboxy isononyl phthalate (MCINP), mono-2-ethyl-5-carboxypentyl terephthalate (MECPTP), mono-2-ethyl-5-hydroxyhexyl terephthalate (MEHHTP), mono (3-carboxypropyl) phthalate (MCPP)), and two metabolites of DINCH (cyclohexane-1,2-dicarboxylic acid mono carboxyisooctyl ester (MCOCH), and cyclohexane-1,2-dicarboxylic acid mono hydroxyisononyl ester (MHNCH)). Of note, DEHP and DEHTP are subsets of the HMW group.

### Primary exposure variables

Individual metabolites were combined into five molar groups: DINCH (MCOCH + MHNCH), Low Molecular Weight (LMW; MEP + MIBP + MNBP), High Molecular Weight (HMW; MBZP + MCINP + MCIOP + MCPP + MECPP + MECPTP + MEHHP + MEHHTP + MEHP + MEOHP + MINP), DEHP (MECPP + MEHHP + MEHP + MEOHP), and di-2-ethylhexyl terephthalate (DEHTP; MECPTP + MEHHTP). Values below the limit of detection (LOD) for individual components were included in molar sum groups. Since urine volumes differ, each metabolite was corrected by specific gravity using the Boeniger formula [16]: Pc = P[(SGm − 1)/(SG − 1)], where Pc is the specific gravity-corrected toxicant concentration (ng/ml), P is the observed toxicant concentration (ng/ml), SGm is the median SG value among the study population, and SG is the specific gravity of the individual urine sample (https://hhearprogram.org/variable-urine-dilution). The specific gravity-corrected metabolite values were converted to moles by dividing by their molecular weight, and then summed to create molar groups. Since the groups had extremely skewed distributions, they were natural log-transformed for regression analysis. The DINCH group had one extreme negative outlier that was excluded for regression analysis.

### Child obesity and body fat

Weight, height, and waist circumference were measured during the child’s clinical assessment. Weight was measured using an electronic scale and height was measured using a stadiometer, with child clothed but shoes removed. Waist circumference was measured against the skin in a horizontal plane to the right ilium at the end of expiration. Measures were done twice, with a third if the measures differed by ≥1 pound, 0.25 inch, or 0.5 cm, respectively. Child age- and sex-adjusted BMI (kg/m^2^) percentiles were calculated using the 2000 U.S. CDC Growth Charts [17]. BMI percentile was categorized as children with normal weight (<85th percentile), overweight (85th–<95th percentile), or obesity (≥95th percentile) [18]. Child age- and sex-standardized waist circumference percentiles were calculated using data from the U.S. NHANES, cycle III [19]. Fat mass and fat-free mass were assessed using the RJL systems Quantum series Bioelectric Impedance Analyzer (BIA; RJL Systems, Inc. Clinton Township, MI US)) [20, 21]. Percent (%) body fat was computed as [fat mass / (fat + fat-free mass) × 100].

### Covariates

Child’s age, sex, and race-ethnicity category as well as maternal education level were obtained from the ECHO-FGS clinical exam based on maternal report. Mothers identified their child’s race-ethnicity as Asian/Pacific Islander, Hispanic, NHB, or NHW. Race and ethnicity were viewed as social constructs. Maternal pre-pregnancy BMI was based on maternal self-report of pre-pregnancy weight and measured height at enrollment into the NICHD—Fetal Growth Studies and was categorized into people with normal weight, overweight, and obesity based on CDC guidelines. Self-reported pre-pregnancy weights and heights were highly correlated with the weights and heights measured during the NICHD Fetal Growth Studies enrollment visit (correlation coefficient *r* = 0.97 for weight; *r* = 0.95 for height). Mothers were asked if they ever breastfed their child and, if so, to estimate breastfeeding duration. Initiation of formula feeding and introduction of anything other than breast milk or formula were also queried. Continuous months of exclusive breastfeeding were derived based on this information and dichotomized at 6 months. The reported activity level of the child was based on the Preschool Physical Activity Questionnaire (Pre-PAQ), a validated instrument for quantitative assessment of physical activity in children [22].

### Statistical analyses

All analyses were done with SAS 9.4 (SAS Institute Inc., Cary, NC, USA), with statistical significance established at *p* < 0.05 for a two-tailed test. Chi-square tests were used to compare each covariate by child BMI category. Kruskal–Wallis tests were used to compare the level of each metabolite and molar group, by child BMI category. A priori decisions based on the scientific literature and plausibility (both biologic and social-economic) determined which covariates were retained in regression models to adjust for confounding (specific gravity, age, sex, race/ethnicity, birthweight, child’s reported activity level, mother’s pre-pregnancy BMI and education, and exclusively breastfeeding for at least 6 months). Uncorrected urinary phthalates and phthalate replacements were included as predictors in regression models and urinary specific gravity was included as a covariate to adjust for differences in urine volume [23].

Each multivariable linear regression model was checked for linearity of the independent variables with the outcome, evidence of multicollinearity, patterns in plots of residuals against predicted values and normal distribution of residuals. Interactions between each of the 5 molar groups and sex as well as between each of the 5 molar groups and race-ethnic group were tested using appropriate interaction terms. When global interaction with *p* < 0.05 were identified, strata specific results are presented. The Durbin–Watson statistic was used to check the model residuals for data independence. Adjustment for multiple comparisons was not done as this is an exploratory study [24, 25].

## Results

### Characteristics of and exposure levels in the study population

**Fig. 1:**
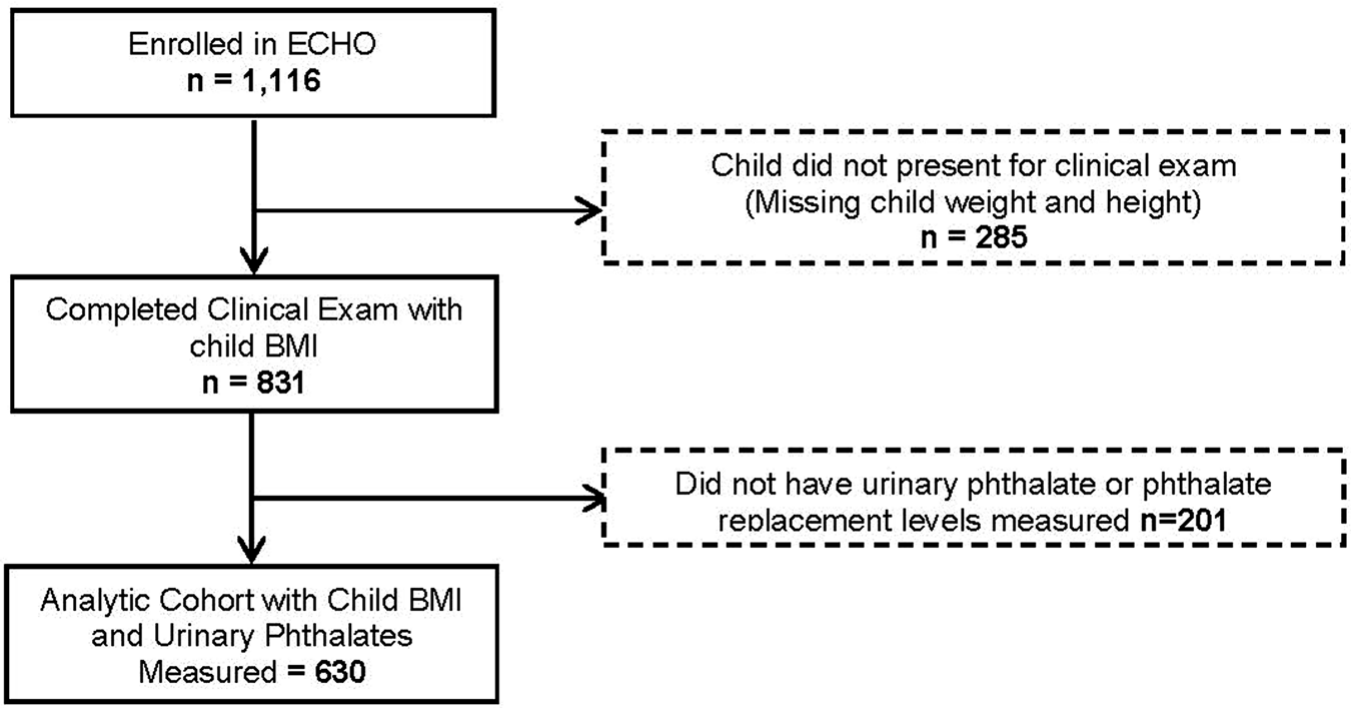
Consort Diagram. Flowchart of ECHO study participants available for analysis of child obesity and urinary phthalate and phthalate replacement levels measured.

In total, 831 children ages 4–8 years completed an in-person ECHO-FGS exam; of these 630 had urinary phthalate and phthalate replacement levels measured and contribute to the current analysis (see Fig. 1). Child mean age was 6.8 years, 48% were female, 26% identified as NHW, 32% identified as NHB, 28% identified as Hispanic and 14% identified as Asian/Pacific Islander (see Table 1). Based on BMI percentiles, children were classified as normal weight (70%), overweight (17%) and with obesity (13%).

**Table 1:** Characteristics of the population for the total population and stratified by child BMI category.

	Total *n* = 630	Normal *n* = 444	Overweight *n* = 107	Obesity *n* = 79	*p* value^a^
Female (*n*, %)	303 (48%)	216 (49%)	53 (50%)	34 (43%)	0.62
Race/ethnicity (*n*, %)					**<0.0001**
Asian/PI	89 (14%)	75 (17%)	11 (10%)	3 (4%)	
Hispanic	175 (28%)	101 (23%)	37 (17%)	37 (47%)	
Non-Hispanic Black	200 (32%)	138 (31%)	33 (31%)	29 (37%)	
Non-Hispanic White	166 (26%)	130 (29%)	26 (24%)	10 (13%)	
Age in years (mean ± SD)	6.8 ± 1.0	6.7 ± 1.0	6.8 ± 1.0	6.9 ± 0.9	0.44
Child’s birthweight (kg), *n* = 606 (mean ± SD)	3.3 ± 0.5	3.3 ± 0.5	3.3 ± 0.5	3.3 ± 0.5	0.42
Child activity level—More active (*n*, %)	286 (63%)	285 (65%)	60 (60%)	41 (53%)	0.08
Exclusively breastfed for at least 6 months (*n*, %)	133 (21%)	110 (25%)	15 (14%)	8 (10%)	**0.002**
Mother’s education—HS or less (*n*, %)	154 (24%)	89 (20%)	33 (31%)	32 (41%)	**<0.0001**
Maternal pre-pregnancy BMI, *n* = 626 (mean ± SD)	25.7 ± 5.4	24.9 ± 5.0	26.7 ± 5.3	28.7 ± 6.2	**<0.0001**

Totals may not equal 100% due to rounding.

*SD* standard deviation.

^a^Comparison of 3 BMI categories by Chi-square or Kruskal–Wallis test. Bold indicates *p* < 0.05.

Median values of each of the 5 molar sum groups, corrected for specific gravity, are found in Table 2 for the total population and stratified by child BMI category. For LMW, HMW, DEHP, and DEHTP molar sum groups, all children had at least one measured component value above the LOD. For the DINCH molar sum group, 88% of children had values of MCOCH and/or MHNCH above the LOD. Median values varied significantly across BMI categories for the LMW molar sum group with median value being 0.37 nmol/l (95% CI: 0.32, 0.48) in children with obesity, 0.26 nmol/l (95% CI: 0.23, 0.32) in children with overweight and 0.26 nmol/l (95% CI: 0.22, 0.29) in children of normal weight. Median values did not differ significantly across child BMI categories for DINCH, HMW, DEHP or DEHTP molar sum groups. Supplementary Table 1 provides parallel information stratified by child race-ethnic group.

**Table 2:** Median values of molar groups, corrected for specific gravity, for total cohort and by BMI categories, *N* = 630.

Molar sum groups	% of tests > LOD	All (*N* = 630) nmol/l (95% CI)	Normal (*n* = 444) nmol/l (95% CI)	Overweight (*n* = 107) ng/ml (95% CI)	Obesity (*n* = 79) nmol/l (95% CI)	*p* value^a^
DINCH	88	0.0040 (0.0037, 0.0045)	0.0040 (0.0036, 0.0046)	0.0035 (0.0026, 0.0044)	0.0046 (0.0037, 0.0056)	0.15
LMW	100	0.27 (0.25, 0.29)	0.26 (0.22, 0.29)	0.26 (0.23, 0.32)	0.37 (0.32, 0.48)	**0.0002**
HMW	100	0.40 (0.36, 0.45)	0.40 (0.36, 0.46)	0.32 (0.29, 0.49)	0.47 (0.36, 0.51)	0.17
DEHP	100	0.12 (0.11, 0.13)	0.12 (0.11, 0.13)	0.11 (0.09, 0.13)	0.14 (0.11, 0.17)	0.06
DEHTP	100	0.19 (0.18, 0.22)	0.20 (0.18, 0.23)	0.16 (0.14, 0.23)	0.20 (0.14, 0.30)	0.56

Bold indicates *p* < 0.05.

DINCH = MCOCH + MHNCH; LMW = MEP + MIBP + MNBP; HMW = MBZP + MCINP + MCIOP + MCPP + MECPP + MEHHP + MEHP + MEOHP + MECPTP + MEHHTP + MINP; DEHP = MECPP + MEHHP + MEHP + MEOHP; DEHTP = MECPTP + MEHHTP.

^a^Comparison of 3 BMI categories by Kruskal–Wallis test.

### Primary linear regression results

Separate multivariate linear regression models were run for each of the 5 molar sum groups in relationship to BMI *z*-score, waist circumference *z*-score and % body fat (see Table 3). Each molar group was natural log-transformed, and all models were adjusted for specific gravity, child sex, age, race/ethnicity, birthweight, reported activity level, mother’s pre-pregnancy BMI and education, and whether child was exclusively breastfed for at least 6 months. A one unit increase in the log of DINCH was associated with a 0.106 unit decline in BMI *z*-score [*β* = −0.106 (95% CI: −0.181, −0.031)], a 0.119 unit decline in waist circumference *z*-score [*β* = −0.119 (95% CI: −0.189, −0.050)] and a 0.012 unit decline in % body fat [*β* = −0.012 (95% CI: −0.019, −0.005)]. LMW, HMW, DEHP and DEHTP were not associated with BMI *z*-score, waist circumference *z*-score, or % body fat in linear regression models examined.

**Table 3:** Multivariate^a^ linear regression results with separate models run for each molar sum group with each outcome.

Main independent variables	Child adiposity/obesity outcomes		
	BMI *Z*-score, *n* = 582, *β* (95% CI)	Waist circumference *Z*-score, *n* = 582, *β* (95% CI)	Percent body fat, *n* = 457, *β* (95% CI)
DINCH	**−0.106 (−0.181, −0.031)**	**−0.119 (−0.189, −0.050)**	**−0.012 (−0.019, −0.005)**
LMW	0.06 (−0.02, 0.15)	−0.003 (−0.08, 0.08)	0.002 (−0.006, 0.010)
HMW	−0.02 (−0.12, 0.07)	−0.01 (−0.10, 0.08)	−0.00004 (−0.009, 0.009)
DEHP	−0.05 (−0.15, 0.06)	−0.05 (−0.15, 0.05)	0.001 (−0.008, 0.011)
DEHTP	−0.02 (−0.09, 0.05)	−0.01 (−0.07, 0.06)	−0.001 (−0.007, 0.006)

The five molar sum groups served as the main independent variables with separate models run for each molar sum group with each outcome. All molar groups are natural log-transformed. DINCH models have one less observation due to dropping one with a negative value. Bold indicates *p* *<* 0.05.

*β* beta estimate, *CI* confidence interval.

^a^All models adjusted for urinary specific gravity, child sex, child age, child race/ethnicity, child birthweight, child’s reported activity level, mother’s education, mother’s pre-pregnancy BMI, and whether child was exclusively breastfed for at least 6 months.

### Sex-specific interactions for linear regression results

Interactions between each of the 5 molar sum groups and sex were examined in relationship to BMI *z*-score, waist circumference *z*-score, and % body fat. No interactions with sex were identified for the molar sum groups DINCH, LMW, HMW or DEHP; however, interactions were identified between sex and DEHTP for the outcomes BMI *z*-score (interaction *p* value = 0.0110) and % body fat (interaction *p* value = 0.0465). There was an inverse relationship between DEHTP and BMI *z*-score in girls [*β* = −0.10 (95% CI: −0.19, −0.008)], but no clear association in boys [*β* = 0.06 (95% CI: −0.03, 0.16)]. There was a nonsignificant inverse relationship between DEHTP and % body fat in girls [*β* = −0.006 (95% CI: −0.015, 0.002)], while there was a nonsignificant positive relationship between DEHTP and % body fat in boys [*β* = 0.006 (95% CI: −0.003, 0.014)].

### Race-ethnic group-specific interactions for linear regression results

Interactions between each of the 5 molar sum groups and race-ethnicity were examined in relationship to BMI *z*-score, waist circumference *z*-score, and % body fat (see Table 4). No interactions with race-ethnic group were identified for BMI *z*-score or waist circumference *z*-score. However, for % body fat, interactions were identified between race-ethnic group and DINCH (*p* value = 0.0031), race-ethnic group and LMW (*p* value = 0.0019), and race-ethnic group and HMW (*p* value = 0.0374). For the DINCH molar sum group there was an inverse relationship between DINCH and % body fat in Asian/PI [*β* = −0.038 (95% CI: −0.054, −0.022)] and in non-Hispanic White individuals [*β* = −0.016 (95% CI: −0.029, −0.004)], but no clear association in Hispanics and non-Hispanic Black individuals. For the LMW molar sum group there was an inverse relationship between LMW and % body fat in Asian/PI [*β* = −0.024 (95% CI: −0.040, −0.007)], but a positive association between LMW and % body fat in NHB [*β* = 0.015 (95% CI: 0.004, 0.026)] with no clear association in Hispanic or NHW individuals. For the HMW molar sum group there was an inverse relationship between HMW and % body fat in Asian/PI [*β* = −0.023 (95% CI: −0.043, −0.002)], but no clear association in Hispanic, NHB, or NHW individuals.

**Table 4:** Multivariate^a^ linear regression estimates in each racial-ethnic group for the relationship between DINCH, LMW and HMW molar sum groups and percent body fat.

	Interaction *p* value	Percent body fat, *N* = 457, *β* (95% CI)
DINCH	**0.0031**	
Asian/Pacific Islander		**−0.038 (−0.054, −0.022)**
Hispanic		−0.008 (−0.021, 0.004)
Non-Hispanic Black		−0.004 (−0.014, 0.006)
Non-Hispanic White		**−0.016 (−0.029, −0.004)**
LMW	**0.0019**	
Asian/Pacific Islander		**−0.024 (−0.040, −0.007)**
Hispanic		0.003 (−0.010, 0.015)
Non-Hispanic Black		**0.015 (0.004, 0.026)**
Non-Hispanic White		−0.002 (−0.016, 0.012)
HMW	**0.0374**	
Asian/Pacific Islander		**−0.023 (−0.043, −0.002)**
Hispanic		0.011 (−0.004, 0.026)
Non-Hispanic Black		0.005 (−0.008, 0.019)
Non-Hispanic White		−0.006 (−0.020, 0.008)

Separate models were used for each molar sum group. Bold indicates *p* < 0.05.

^a^All molar sum groups were natural log-transformed. All models adjusted for urinary specific gravity, child sex, child age, child race/ethnicity, child birthweight, child’s reported activity level, mother’s education, mother’s pre-pregnancy BMI, and whether child was exclusively breastfed for at least 6 months.

### Multinomial logistic regression results

Finally, separate multinomial logistic regression models were run for each of the 5 molar sum groups to compare the odds of having overweight or obesity compared to normal weight (see Table 5). All models were adjusted for the covariates previously noted. Molar sum groups DINCH, HMW, DEHP and DEHTP were not associated with children having overweight or obesity. The LMW sum group was not significantly associated with odds of being overweight [OR = 1.11 (95% CI: 0.85, 1.42)], but was significantly associated with an increased odds of obesity [OR = 1.35 (95% CI: 1.02, 1.79)].

**Table 5:** Multinomial^a^ model for the outcomes of children having overweight and obesity compared to normal weight.

Main independent variable	Child adiposity outcomes (*N* = 582)	
	Overweight OR (95% CI)	Obesity OR (95% CI)
DINCH	0.83 (0.66, 1.05)	0.83 (0.63, 1.08)
LMW	1.11 (0.85, 1.42)	**1.35 (1.02, 1.79)**
HMW	0.77 (0.57, 1.04)	1.19 (0.86, 1.64)
DEHP	0.78 (0.56, 1.08)	1.11 (0.77, 1.59)
DEHTP	0.88 (0.71, 1.10)	1.06 (0.83, 1.36)

Separate models were run for each of the five molar groups which served as the main independent variables.

All molar groups are natural log-transformed. One observation was dropped from DINCH models due to dropping one with a negative value. Bold indicates *p* < 0.05.

*OR* odds ratio, *CI* confidence interval.

^a^All models adjusted for urinary specific gravity, child sex, child age, child race/ethnicity, child birthweight, child’s reported activity level, mother’s education, mother’s pre-pregnancy BMI, and whether child was exclusively breastfed for at least 6 months.

## Discussion

In this cross-sectional investigation of children’s urinary phthalates and adiposity in a racially/ethnically diverse US population, we found a consistent association between higher DINCH levels and lower BMI *z*-score, waist circumference *z*-score, and % body fat. Moreover, we report an interaction between race-ethnic group, DINCH exposure, and % body fat, with a strong inverse association identified in Asian/PI children and a slightly weaker inverse association identified in NHW children. However, analyses that categorized BMI into normal, overweight, and obesity categories were null for the DINCH molar sum group. We did not find evidence of sex-specific effects for DINCH, LMW, HMW or DEHP molar sum group exposures in relationship to any of our outcomes. However, we did identify interactions between sex and DEHTP for the outcomes BMI *z*-score (interaction *p* value = 0.0110) and % body fat (interaction *p* value = 0.0465). There was an inverse relationship between DEHTP and BMI *z*-score in girls [*β* = −0.10 (95% CI: −0.19, −0.008)], but no clear association in boys [*β* = 0.06 (95% CI: −0.03, 0.16)]. Although nonsignificant there was an inverse relationship between DEHTP and % body fat in girls, and a positive relationship between DEHTP and % body fat in boys.

Unique aspects of our study include its diverse population, extensive characterization of both maternal and child factors and our inclusion of DINCH, a phthalate replacement that has not received a great deal of attention or regulation. Further, our examination of race-ethnicity as a potential modifier of the relationship between phthalates and phthalate replacements and child markers of adiposity is novel. Moreover, few prior studies have included the outcome child % body fat and few studies have focused on or included children ages 4–8 years [6, 8].

Exposure to phthalates and phthalate replacement chemicals was practically ubiquitous in our diverse study population, with 88% exposed to at least one DINCH metabolite and all children exposed to LMW and HMW phthalate metabolites. Neither LMW nor HMW exposure was significantly associated with BMI *z*-score or waist circumference *z*-score. Analyses that categorized BMI into normal, overweight, and obesity categories were also largely null for the LMW and HMW molar sum groups, except for an increased odds of obesity with increasing levels of the LMW molar sum group. In contrast to prior studies of children’s phthalates we did not find evidence of sex-specific effects for LMW, HMW, or DEHP molar sum groups in association with our outcomes of interest [6–10]. However, interactions were identified between sex and DEHTP for the outcomes BMI *z*-score (interaction *p* value = 0.0110) and % body fat (interaction *p* value = 0.0465). Moreover, we found evidence of interactions between race-ethnic groups and phthalates for the outcome of % body fat. There was an inverse association identified in Asian/PI children and a positive association in NHB children between LMW phthalate levels and % body fat. Similarly, in Asian/PI children we found an inverse association between HMW phthalate levels and % body fat, but no other significant associations in the other race-ethnic groups. A possible explanation of why NHB children had positive associations between LMW phthalate levels and % body fat could be due to the use of ethnic personal care products known to contribute to higher phthalate exposures. A pilot study on occupational exposures to phthalates among Black and Latina hairdressers with diverse customers reported higher urinary metabolite concentrations compared to office worker control subjects. The geometric mean for MEP was 10 times higher in the hairdressers (161.4 ng/ml) compared to the control (15.3 ng/ml) [26]. Further studies are needed to characterize such exposures and inform regulatory decisions that contribute to health disparities among vulnerable populations.

Phthalates are known for their endocrine disruption properties, meaning that they can interfere with the natural process that the endocrine hormone system accomplishes [27]. The mechanisms underlying the endocrine disruption properties of phthalates and their potential relationship to BMI and waist circumference remain unknown. Studies indicate that phthalate metabolites may act as obesogens through several mechanisms including epigenetic modulation, activation of peroxisome proliferator-activator receptors (PPARs) and antithyroid effects [11, 28–32]. DINCH is a non-phthalate plasticizer that has replaced DEHP in several applications. While early toxicologic animal studies indicated that DINCH had no relevant endocrine disruption properties with respect to obesogenic effects, and a weaker effect on PPARα than DEHP [33, 34], recent studies indicate that DINCH may have more significant effects than initially assumed [35–37]. Focusing on potential obesogenic mechanisms, DINCH and its metabolite monoisononylcyclohexane-1,2-dicarboxylic acid ester (MINCH) have been shown to induce oxidative stress and enhance inflammatory responses in human THP-1 macrophages [37], establishing a potential link since macrophage dysfunction is associated with obesity [38]. A recent in vitro study also reported that MINCH accelerated human adipogenesis through PPARγ and caused oxidative stress and metabolic homeostasis in mature adipocytes [36]. Only a limited number of recent studies have examined associations between DINCH and adiposity/obesity with few focused on children [4, 39, 40]. Pacyga et al. found some evidence of an inverse association between gestational weight gain and urinary DINCH [41]. While Correia-Sa et al. found no difference in DINCH levels between two groups of lighter and heavier children, the study had a significant limitation in that the heavier children were being prescribed a diet based on fresh food with less packaged and processed foods [40].

Our study has several strengths including knowledge gaps addressed. Recently, reviews and meta-analyses have been published focusing on the association between exposure to phthalates and child adiposity/obesity measures [29, 42, 43]. One meta-analyses examined 13 individual phthalate metabolites in association with child/adolescent BMI, BMI *z*-score and waist circumference, while the second focused on a total of 10 phthalate metabolites and included child BMI, BMI *z*-score, obesity and waist circumference as outcomes of interest [29, 42]. Results from both meta-analyses indicated that associations reported in children have been in both directions with many null associations [29, 42]. Additionally, sex-specific differences were inconsistent across studies [6–10]. While some studies did include diverse cohorts, such as studies conducted in NHANES, race-ethnic specific differences have not previously been reported. While a single study indicated different associations based on age of exposure, most studies in children have not been powered to examine associations in children ages 4–8 years. Finally, very few studies have examined child adiposity/obesity markers other than BMI or waist circumference, such as % body fat.

Limitations of our study should also be noted. Our study is one of the largest to describe a racially/ethnically diverse population of children to date, yet the smaller sample sizes within subgroups limited our ability to fully examine race-ethnic specific effects or sex-specific effects. Future studies will need to validate our reported race-ethnic specific effects. We were also limited in that we did not have information on child diet or a direct measure of child physical activity level. Our study is also limited by its cross-sectional design, and so it is unclear if children with greater adiposity are exposed to or retain less DINCH than children with less adiposity. Large longitudinal studies with serial measures of phthalate levels and adiposity throughout childhood, enabling the examination of temporal relationships between phthalate levels, phthalate replacements, and child adiposity are needed.

We used an LC-MS/MS based method to determine urinary phthalate concentrations, a preferred approach because of its high selectivity and sensitivity without the derivatization step required of gas chromatography. We implemented U.S. CDC quality control standards to ensure the measurement validity [15], and we adjusted for urinary specific gravity to accommodate differences in urinary volume from the spot urine specimens. However, the short half-life of urinary phthalates increases variability in measured phthalate levels and potentially introduces exposure measurement misclassification. A recent review suggested good reliability for single urinary measures of LMW phthalates in adults (intraclass correlation coefficient (ICCs) > 0.4), but poor reliability for HMW phthalates (ICCs < 0.4) [44]. Studies of children followed a similar pattern, but with lower ICCs. However, a single urinary phthalate measure reliably predicted 6-month mean urinary phthalate concentration rankings in young children, suggesting the validity of single urinary phthalate specimens to estimate longer term exposure [45]. Still, our results may underestimate the population-level risks due to exposure measurement error. A future investigation with multiple urine specimen collections over several months will be necessary to confirm our results. Additional research is also needed to identify possible interactions between exposure to LMW phthalates, HMW phthalates, phthalate replacements, and child adiposity/obesity.

In summary, our study contributes to the body of literature examining the association between child exposure to phthalates and child adiposity/obesity levels and begins to close some of the knowledge gaps. It is one of the first studies to also include DINCH, a phthalate replacement, and we report a consistent inverse association between DINCH and markers of child adiposity/obesity. While we also report a few sex-specific and race-ethnic-specific findings for phthalates and DINCH, most of our analyses indicated null associations between phthalates and adiposity/obesity outcomes. Given that these chemicals are ubiquitous in the environment and have known endocrine disrupting properties, our understanding of their relationship to adiposity/obesity may shed light on the ongoing child obesity epidemic.

### Supplementary information

Supplemental Table 1.

## Data Availability

The datasets generated during and/or analyzed during the current study are not publicly available because a data use agreement is required for their use but are available from the corresponding author on reasonable request if a data use agreement is established.
