# Supplementary material for: Phthalate and phthalate replacement concentrations in relationship to adiposity in a multi-racial cohort of children
Source: Int J Obes (Lond). 2024 Jun 1;48(9):1266–73. doi: 10.1038/s41366-024-01548-w (PMC11347365; doi:10.1038/s41366-024-01548-w)
Supplement: Supplementary file 1 — Supplemental Table 1. [file 41366_2024_1548_MOESM1_ESM.docx]

**Supplemental Table 1. Median values of molar groups, corrected for specific gravity, for total cohort and by race/ethnic group, N=630.**

| **Molar Sum Groups** | **% of tests >LOD** | **All (N=630)**  **nmol/L (95% CI)** | **Non-Hispanic White (n=166) nmol/L (95% CI)** | **Asian/PI (n=89)**  **ng/ml (95% CI)** | **Hispanic (n=175)**  **nmol/L (95% CI)** | **Non-Hispanic Black (n=200) ng/ml (95% CI)** | ***p*-value*** |
| --- | --- | --- | --- | --- | --- | --- | --- |
| **DINCH** | 88 | 0.0040 (0.0037, 0.0045) | 0.0033 (0.0029, 0.0037) | 0.003 (0.002, 0.004) | 0.0046 (0.0040, 0.0054) | 0.005 (0.004, 0.007) | **<.0001** |
| **LMW** | 100 | 0.27 (0.25, 0.29) | 0.19 (0.16, 0.21) | 0.26 (0.19, 0.29) | 0.28 (0.24, 0.32) | 0.39 (0.35, 0.45) | **<.0001** |
| **HMW** | 100 | 0.40 (0.36, 0.45) | 0.34 (0.30, 0.39) | 0.33 (0.30, 0.43) | 0.40 (0.34, 0.48) | 0.58 (0.49, 0.66) | **<.0001** |
| **DEHP** | 100 | 0.12 (0.11, 0.13) | 0.09 (0.08, 0.11) | 0.12 (0.10, 0.14) | 0.13 (0.11, 0.15) | 0.14 (0.13, 0.17) | **<.0001** |
| **DEHTP** | 100 | 0.19 (0.18, 0.22) | 0.16 (0.14, 0.19) | 0.16 (0.12, 0.22) | 0.16 (0.14, 0.20) | 0.27 (0.23, 0.34) | **<.0001** |

*Comparison of 4 race/ethnic groups by Kruskal-Wallis test.

**DINCH**=MCOCH + MHNCH

**LMW**=MEP + MIBP + MNBP

**HMW**=MBZP + MCINP + MCIOP + MCPP + MECPP + MEHHP + MEHP + MEOHP + MECPTP + MEHHTP + MINP

**DEHP**=MECPP + MEHHP + MEHP + MEOHP

**DEHTP**=MECPTP + MEHHTP
